# Cross-cultural differences in early caregiving: levels of mind-mindedness and instruction in UK and India

**DOI:** 10.3389/frcha.2023.1124883

**Published:** 2023-08-14

**Authors:** Laura Bozicevic, Jonathan Hill, Prabha S. Chandra, Agni Omirou, Chaithra Holla, Nicola Wright, Helen Sharp

**Affiliations:** ^1^ Department of Primary Care & Mental Health, Institute of Population Health, University of Liverpool, Liverpool, United Kingdom; ^2^ School of Psychology and Clinical Language Sciences, University of Reading, Reading, United Kingdom; ^3^ National Institute of Mental Health and Neurosciences, Bangalore, India; ^4^ Department of Psychology, Manchester Metropolitan University, Manchester, United Kingdom

**Keywords:** parenting, cross-culture, observational method, mother–child interaction, mind–mindedness, instructions, parental control, warmth

## Abstract

**Introduction:**

Most studies on parenting and its role in child development are conducted in Western countries, but it cannot be assumed that characteristics of parental practices are similar in non-Western settings. Research characterizing cultural differences in parenting is required to inform the focus of studies designed to test differential outcomes from such practices in children over time and across cultures. The present cross-cultural study examined differences in maternal speech during mother–child interactions, and, specifically, in the use of mind-mindedness, instruction and control, and the expression of warmth (i.e., positive comments).

**Methods:**

We observed 100 dyads (50 from the UK and 50 from India) during mother-infant play interactions at 7 months. Maternal speech was transcribed and translated prior to independent coding, and this was coded using established measures together with a newly developed measure of “Instructions”.

**Results:**

Substantially large differences between UK and Indian mothers were observed. Compared with UK mothers, Indian mothers made fewer mind-minded comments about their infants, and they issued more instructions and made more controlling and positive comments. Findings from this study might reflect cultural differences in how parental style might be expressed according to cultural priorities and values.

**Conclusions:**

The implications of these very large differences in parenting across cultures for child development remain to be investigated and are discussed in the present paper.

## 1. Introduction

Parenting style characterized by responsiveness to infants’ behaviors and likely mental states is associated with better emotional and behavioral outcomes during childhood and beyond (1, 2). This understanding of the role of early parenting is mainly based on research conducted in Western settings and the associations between these parental practices and child outcomes cannot be assumed to be universal (3–5). In fact, there may be cultural variations in norms and values that lead non-Western parents to prioritize different behaviors, such as providing guidance to their children, and these may be evident even during infancy (3, 4). In turn these culturally-valued parental behaviors may be associated with favorable outcomes in these settings. The extent and types of cultural differences in parental practices are inconsistently explored in research; their investigation is important because it might contribute to the further understanding not only of child development in non-Western settings, but also of cultural variations in the relationship between parenting and child outcomes vs. “universal” associations which can be found across settings (5). In this study, we analyzed maternal speech during a standardized play procedure between UK and Indian mothers with their infants. Based on available evidence on differences in parenting practices we examined the hypothesis that Indian mothers, as representative of Asian culture, show more evidence of guidance (i.e., instructions), and they interpret and verbalize infant mental states less often than UK mothers, representative of Western culture.

In recent years a growing body of research has examined the construct of “mind-mindedness”, the caregivers ability to interpret the mental states underpinning their infant's behaviors, which can be assessed as the number of appropriate mind-minded comments made during interactions with their children (6). There is a wealth of data supporting the idea that early parenting in which the parent seeks to follow and understand their infant's behaviors, is associated with a wide range of positive cognitive, behavioral and social outcomes. In fact, higher levels of mind-minded comments have been associated with better social-emotional understanding (7, 8) and secure attachment (9–11), and fewer behavioral problems and emotional difficulties (10, 12, 13). These findings are however mainly drawn from Western research (2, 14) with the question of whether they are cross-cultural processes yet to be answered. As theoretical formulations link mind-mindedness to the promotion of autonomy and self-expression (independent qualities) in children (15), mind-mindedness might be seen as less relevant and therefore displayed less by parents from cultural settings that value interdependent qualities (e.g., obedience, respect for elders) more than independent qualities. This has been supported in a few cross-cultural studies: three found that Chinese mothers of eighteen months-three years old children are less mind-minded than US, UK and Australian mothers during a story-telling task, when they are asked to talk about their children, and during an observed play interaction (16–18). Moreover, a study comparing Japanese and British mothers when talking about their 3–6 years old children found that Japanese women made a significantly lower proportion of mind-related comments compared to British women (19). Finally, in a comparison of 29 German and 28 Indian infants aged 3 months, Keller and colleagues (2010) reported higher levels of the broad construct “autonomy promoting” behaviors in the German families, and higher levels of “relatedness” in the Indian families (20). Autonomy promoting included scales similar to mind-mindedness, and relatedness included references to social norms. Therefore, to date, there are only a few existing cross-cultural studies assessing the construct of mind-mindedness in Asian settings, and even fewer in South Asian settings, and no previous study has compared parenting across cultural settings using blind ratings to deal with potential rating bias based on cultural expectations.

Another widely observed characteristic of parenting is guiding children to teach them appropriate behaviors, and social, cognitive and motor skills. Although this parental practice might be observed across cultures, studies conducted in non-Western settings suggest that parents from cultures which value interdependent qualities (e.g., obedience), such as Asian populations, may give particular priority to parental behaviors aimed to guide their children, such as teaching and instructing, rather than respond to their needs and verbalize their mental states (16, 21). There is, however, limited evidence regarding these parental practices in infancy. For instance, Reddy and colleagues compared parent-infant interaction during daily life between 6 and 12 months of age in nine middle-class urban families in the United Kingdom and thirteen middle-class urban families in India; they found that rates of parent directives to the infants were higher in the Indian dyads (22, 23). Directives were characterized as either positive, for example “Press this one” or “You try it” and negative, for example “No, don't go” or “Don't put it in your mouth.” While these studies are notable for many reasons (e.g., early assessment of directiveness, use of observational methods, cross-cultural comparison), the sample sizes were small, and families included belonged to the urban middle class. While telling children what to do to regulate their behavior it is seen as desirable in non-Western settings as it promotes obedience and respect for elders as well as appropriate behavior, these practices might be perceived as controlling (i.e., intrusive, pressurizing, or dominating) in North American and European settings as they undermine children's sense of autonomy (24, 25). Although “positive” forms of control do exist (e.g., authoritative practices including parental guidance, monitoring, and rule setting), high levels of behavioral control, assessed through observations of Western parents interacting with their children, have predominantly been associated with poorer developmental outcomes including insecure attachment (26, 27), and externalizing and internalizing problems (28–30). A key question therefore is whether parental instructions and parental behavioral control are essentially the same (e.g., parental guidance), but valued to different degrees across cultures, and perhaps also associated with different developmental outcomes, or whether they are different constructs. In this study, we set out to examine cross-cultural use of parental “Instructions” (assessed using a newly developed scale) and we also coded parental “Control” using a European measure in order to compare the two.

Maternal warmth or positive affect (i.e., affection and acceptance expressed toward children) is considered another key parenting dimension which promotes child adjustment (e.g., secure attachment, fewer internalizing and externalizing problems, and fewer callous-unemotional traits) and optimal cognitive, and social-emotional development (5, 28, 29, 31–33). Parental positive affect appears to be associated with mind-mindedness, in Western populations (13, 32), and with control in Asian settings (34). These results might indicate that positive affect is a common factor among different cultures associated with good outcomes irrespective of other dimensions of parenting considered desirable in different cultures (5). It may also moderate the association between parental control and negative child outcomes, such as externalizing and internalizing problems (35, 36).

The main aim of the study was to compare maternal speech in a sample of UK and Indian mother-infant dyads while interacting in a play-based task, in their levels of mind-mindedness, instructions, control and positive affect. We hypothesized that Indian mothers would use parental practices aimed to guide the children as evidenced by higher levels of instructions to a greater extent than mothers from UK, who, by contrast we hypothesized would use practices aimed to understand their infants’ motives as evidenced in higher numbers of mind-minded comments. We did not make predictions for the direction of any differences in levels of control, nor of positive affect across Indian and UK families.

## 2. Materials and methods

### 2.1. Procedure and design

**Figure 1:**
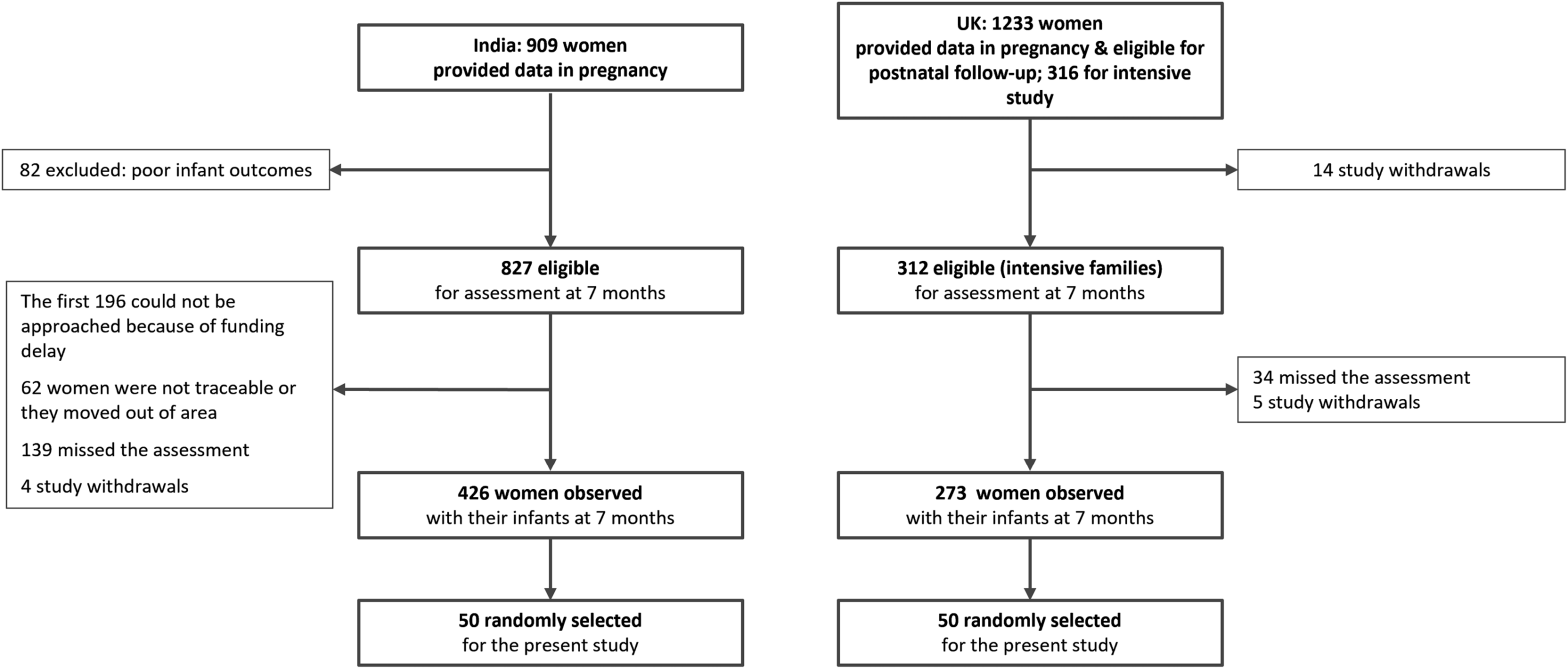
Flow chart showing the selection of the sample.

Participants were identified from two longitudinal studies in the UK and India with planned common measurement at parallel time points. In both studies only women aged 18 or above, who gave birth to a live singleton baby, without severe congenital abnormality were included in the samples. The UK Wirral Child Health and Development Study (WCHADS) sample has been described in previous publications (37). In brief, first time pregnant women, who could speak English, were recruited at 20 weeks from a publicly funded (NHS) maternity unit serving a defined geographical area with a broad representation of socioeconomic conditions but very few non-White inhabitants. WCHADS is representative of the population of child-bearing age women in the area from which the sample was drawn, the Wirral (UK), which is slightly more deprived than the rest of the UK. From a total of 1,233 women recruited, 316 were selected for intensive study, stratified by psychosocial risk, and of them 273 were observed with their infants at 7 months (Figure 1). In India participants were drawn from the Bangalore Child Health and Development Study (BCHADS) who were first recruited into the Prospective Assessment of Maternal Mental Health Study, PRAMMS (38) and then followed up through infancy in the BCHADS. In the PRAMMS 909 women attending the Antenatal clinic at the Government Referral Hospital (GRH) in South Bangalore (India) were recruited during the first two trimesters of pregnancy [see (36) for further information on the recruitment]. Exclusion criteria included having a major mental illness, having had major health complications during the current pregnancy, reporting harmful use of alcohol or other psychoactive substances, not speaking the language for assessment (Kannada), and not planning to reside in the same city. Women were from predominantly from low-income groups. From the original sample, 825 women had a singleton and live birth and were eligible for postnatal assessments and, of them, 407 were observed with their infants at 7 months (Figure 1). Compared to the population of the Karnataka urban areas, the BCHADS cohort is similar in terms of education, and marital and socio-economic status (39, 40).

Informed consent was obtained from parents in both studies. The UK study was approved by the Cheshire North and West Research Ethics Committee (UK) on the 27th of June 2006. The Indian study was approved by the National Institute for Mental Health and Neuroscience (NIMHANS) Ethics Committee on the 2nd July 2015 and the University of Liverpool Ethics Committee (1st March 2016).

### 2.2. Participants

A sample of 100 mothers (50 from UK and 50 from India) was randomly drawn from those with recordings of interactions between mothers and infants in the two studies, with minimization by infant gender. As shown in Table 1, the infants in the Indian sample were on average one month older than in the UK sample, and the Indian mothers were substantially younger than the UK mothers, and were more likely to have left full time education at age 18 or younger which can be expected due to population statistics. In contrast to the UK mothers, all of the Indian mothers were married. The equal numbers of male and female infants, and the differences is parity were by design. It was not feasible to match the samples on income because of the large economic disparities between the countries. According to the World Bank, average annual per capita income in India in 2020 was 1,663 US dollars compared to 36,248 US dollars in the UK. However, both study samples from which the subsample was drawn reflected similar strata of the general populations. In the Indian sample 50% of the families with available data had an “upper-low” income which corresponds to a monthly income of 12,000 Rs (£121.37) (37). In the UK sample we characterized families using the Index of Multiple Deprivation (38) which uses postal codes to assign codes based on area income, employment, health, education and training, barriers to housing and services, living environment and crime. Based on this classification 54% of the UK families were living in postcodes characterized nationally as belonging in the most deprived quintile.

**Table 1:** Demographic characteristics of the UK and Indian families.

	UK (*N* = 50)	India (*N* = 50)	*p*-Value
Infant's age (months)	6.8 (sd 0.73)	7.8 (sd 1.56)	<.001
Infant's gender (male)	50 (50%)	50 (50%)	
Maternal age (years)	27.3 (sd 6.28)	22.8 (sd 2.64)	<.001
Education finished beyond 18 years	23 (46.0%)	5 (10.0%)	<.001
Parity (primiparous)	50 (100%)	25 (50%)	
Marital status (married/cohabiting)	31 (62.0%)	50 (100%)	<.001

Values of *p* are provided for comparisons of means using independent groups *t*-tests, and for comparisons of binary variables using *χ*^2^. No formal comparison tests were conducted for infant gender and parity as they were determined by the study design.

### 2.3. Measures

#### 2.3.1. Maternal speech during mother-infant interactions

All of the mother-infant interactions in the UK were recorded at a community centre in rooms designed for filming, while in India 40% were filmed in a similar setting and 60% at home. Mothers from both samples were filmed in a 15 min dyadic free play interaction with their children following the procedure developed by the National Institute of Child Health and Human Development (41). In this procedure mothers are asked to play as they would normally do with their children for 7 min with a child's favorite toy brought from home (which could be an object as well as a toy) and for the following 8 min with a standard set of toys (which were culturally adapted to the two settings). Video-recording mothers and infant while interacting has been demonstrated to be a feasible methodology in a sample of Indian families in a urban setting (42). Moreover, the use of the play-based task with toys was piloted with local mothers prior to starting the study and it appeared to be culturally acceptable; belonging to an urban area along with the presence of local Government Anganwadi Centers which encourage use of play may have facilitated the exposure of families to playing with toys.

Transcripts were made from audio-recordings of mothers’ speech over the 15 min of play with their infants. Indian transcripts were translated into English by members of the team proficient in both languages. All the transcripts were then checked by other members of the team who substituted the cues and references that might have provided evidence of their country of origin (e.g., London bus, Iggle Piggle, chapati) with neutral words which carried similar meaning and would not alter the coding of the comment (e.g., bus, doll/teddy bear, bread). Prior to coding, utterances (i.e., comments) were marked out on each transcript so as to obtain a total number of utterances for each mother; each utterance was coded for the presence of the mutually exclusive codes described below as present or absent. A total number of utterances coded as mind-mindedness, control, instructions, and positive affect was calculated for each woman, providing them of four different total scores. Finally, the percentage of codes for each category out of the total number of utterances was calculated for each mother (i.e., percentage of comments coded as mind-mindedness, percentage of comments coded as instructions, percentage of comments coded as control, and percentage of positive comments) in order to compare them despite differences among women in the length of the speech. These percentages were used in the analyses.

#### 2.3.2. Maternal mind-mindedness

Mind-mindedness was rated on the basis of comments on child's mental states such as desires and preferences (e.g.,: “Do you want to sit?”, “You like the bee, don't you?”), cognition (e.g.,: “Are you more interested in the cube?”, “You know that song”, “Are you ignoring mummy?”), emotions (e.g.,: “Are you getting fed up?”, “You had enough”, “Now you are happy to lie down and play”) or intentions (e.g.,: “You are trying to stand up”, “Are you trying to eat it?”) (43). The use of transcripts meant that appropriateness of mind-minded utterances could not be rated as in previous observational studies of parents and children (2); therefore, only an index of the use of the mind-related comments can be drawn from our results. Percentages of comments coded as mind- mindedness have however been used in previous studies using interviews of parents in which appropriateness cannot be judged because infant behaviors are not being observed (2, 13).

#### 2.3.3. Maternal instructions

In order to clarify whether Indian and UK mothers differ in the extent to which they tell their infants how to behave, we devised an “Instructions” measure to index parental guidance and a coding manual was created *ad hoc* for this study.

The procedure used to generate the Instructions measure and assess inter-rater reliability was as follows. After creating coding rules, ten transcripts from the UK and India, but not included in the present study, were read in order to generate a dictionary of examples and add detail to the rules. A further 10, not included in the final sample, were rated for practice using the coding system and the dictionary and discussed by the research team. Finally, a further 20 transcripts, which were included in the study, were rated by two independent coders to generate percentage scores in relation to the total number of utterances over the assessment period (see Reliability section).

In the coding of Instructions only utterances that unambiguously gave directions regarding the infant's behavior were counted (i.e., mothers indicate an action and the object/way with which to perform the action). Examples included “Get the ball from the box and give it to me”, “Don't put that in your mouth” and “Look at these toys”.

#### 2.3.4. Maternal control

Coding of maternal verbal control was based on a mother–child play coding scheme devised for coding from observations (44, 45). In the original Stein and colleagues’ coding scheme ‘strong control’ includes commands, strong requests, inhibitions, forbids, cautioning, and correcting comments; some of these comments could be coded as both instructions as well as other controlling utterances (e.g., get the ball). However, in order to remove item overlap, the maternal verbal control measure used in this study included all the controlling utterances, but not the comments that were coded as Instructions. Examples of controlling comments included are “No”, “Look”, “I will take it away” “That's not right”, and “You don't need that”.

#### 2.3.5. Maternal positive comments

Coding of maternal positive comments was based on a mother–child play coding scheme devised for coding from observations (44). In this coding scheme complimentary and affectionate comments on children's behavior, character or appearance, such as “What a good boy”, “Well done”, “Clever girl” and “My beautiful baby”, are coded for positive affect.

### 2.4. Reliability

Two trained coders independently coded 20 transcripts and their interrater reliability for maternal speech codes was high for each parenting dimension: mind-mindedness ICC = .98, instructions ICC = .95, control ICC = .99, and positive comments ICC = .91.

### 2.5. Data analyses

Log transformations were applied to all skewed variables, which in each case yielded distributions appropriate for parametric analyses. Group differences in demographic characteristics were analyzed using Chi square and *t*-tests. Bivariate associations between demographic and parenting variables were examined using partial correlation coefficients controlling for membership of UK or Indian groups. Group differences in parental practices were analyzed using transformed percentage scores for each of the parenting dimensions as dependent variables and using Multivariate Analysis of Covariance (MANCOVA); the analysis was performed controlling for maternal age, whether mothers left education at 18 or under, and child age at the time of the assessment. Family income and marital status showed no variability in the Indian families, and parity showed no variability in the UK sample, by design, so these were not included in the analyses.

## 3. Results

From the results it emerges that, on average, Indian mothers made 269.98 (SD: 152.138; range: 31–764) comments, and UK mothers made 191.76 (SD: 72.958; range: 26–334) comments. The Indian mothers spoke more than the UK and so the groups were compared using percentages of the total. Table 2 shows the differences in percentages, which in each case reflected a difference in absolute numbers of comments (see Supplementary Table S2).

**Table 2:** Group differences in the parenting dimensions.

	UK (*N* = 50)		INDIA (*N* = 50)		*F* (1, 95)	*p*
	*M*	SD	*M*	SD		
Mind-minded comments	5.94	3.092	1.99	2.477	38.222	<.001
Instructions	10.88	5.411	28.23	15.314	40.884	<.001
Control	4.94	4.615	24.32	14.382	46.587	<.001
Positive comments	2.39	2.574	13.02	9.687	98.767	<.001

Multivariate Analysis of Covariance (MANCOVA) controlling for maternal age, whether mothers left education at 18 or under, and child age at the time of the assessment.

### 3.1. Correlations among socio-demographic variables and parenting dimensions

As shown in Table 3 older mothers had somewhat lower percentage scores for instructions and verbal control, and a higher percentage of positive comments, although all of the correlations were non-significant. Percentage mind-minded comments were positively associated with higher instructions and positive comments. Although percentage instructions and control utterances were positively correlated, the association was non-significant, suggesting that they measure different aspects of parenting (see Supplementary Table S1 for correlations between parenting dimensions within each group).

**Table 3:** Partial correlation coefficients among socio-demographic variables and transformed parenting dimensions.

	Infant age	Maternal age	Age finish education	Mind-minded comments	Instructions	Control
Maternal age	.040					
Age finished education	−0.086	0.168				
Mind-minded comments	−0.043	0.001	0.065			
Instructions	0.051	−0.144	−0.025	0.211*		
Control	0.011	−0.145	0.060	0.056	0.143	
Positive comments	0.002	0.125	0.047	0.214*	0.164	−0.001

Partial correlations controlled for membership of UK vs. Indian groups.

* *p* < .05.

### 3.2. Group differences in the parenting dimensions

Mean percentage scores for mind-minded comments, positive comments, control and instructions comparing UK and India mothers are shown in Table 2. The untransformed values are shown as they are more readily interpretable than the transformed ones. Values of *F* were derived using transformed scores in MANCOVA controlling for infant age, mother age, and age left education. The model confirmed what is evident in Table 2, that UK mothers make substantially more mind-minded comments than Indian mothers (*d* = .36, *p* < .001), and that Indian mothers utter more instructions (*d* = 1.51, *p* < .001), use more verbal control (*d* = 1.81, *p* < .001), and they make more positive comments (*d* = 1.50, *p* < .001).

## 4. Discussion

### 4.1. Differences in parenting dimensions between UK and Indian mothers

In a comparison of two groups of mothers and infants randomly drawn from general population samples, our hypotheses that UK mothers would make more mind-minded comments, and issue fewer instructions than Indian mothers were confirmed. Although we made no predictions regarding positive affect or levels of verbal control, Indian mothers made considerably more positive and controlling comments than the UK mothers. In each comparison the differences were very large, with differences in transformed scores all over one standard deviation.

These findings suggest that the parenting environments for infants in the UK and in India are markedly different. As we noted earlier, few previous studies have examined these differences using content of speech drawn from observations of parenting. In those that have done so, the differences have been similarly large. The study by Reddy and colleagues (2013) compared guidance assessed as parental “directives”, observed at home in UK and Indian middle-class families. Even in this small sample (*N* = 22) the difference between the groups at 6 months was highly significant, and this reflected a large difference, with UK mothers giving a mean of 12 directives per hour and the Indian mothers, 37 per hour. In the comparison of middle-class German and Indian families by Keller and colleagues (2010) which provides a comparable study of mind mindedness (named “mental states of the baby”) assessed among the indicators of an “autonomy supporting conversational style”, the difference in means was highly significant, with the German sample talking more frequently about children's mental states compared to the Indian sample, suggesting a large difference in a moderately small sample (*N* = 57). These results seem in line with the idea that promoting individuality and autonomy, which is an important socialization goal for parents from Western independent settings, may not have the same central role in non-Western interdependent cultures. These societies place high importance on relatedness with the social group (Keller et al., 2010), and they expect children to incorporate values of obedience, respect for the elders, and conformity to parents’ directives as well as self-control (3, 46).

### 4.2. Potential association between parenting dimensions and child outcomes

The large differences that we found, which seem to be consistent with previous research, pose major questions for our understanding of the role of parenting in early development. If quality of parenting early in development has a crucial role in influencing social, emotional, behavioral, and educational outcomes, then either infants in one setting are receiving markedly less favorable parenting than infants in the other, or the dimensions of parenting associated with positive outcomes are different in the UK and in India. Based on available evidence regarding the prevalence of child psychiatric disorders across the two countries, which are broadly similar, the first interpretation seems unlikely (47, 48). Moreover, any differences are much smaller than would be predicted if the large differences in parenting styles that we found reflected large differences in environmental quality. In addition, data on educational outcomes in the UK, where one of the largest minority groups is Indian, shows that, in primary school, Indian children perform on average better than White British peers (49). Therefore, it is likely that all infants need sensitive and attentive parenting, but this is manifested differently across cultures and that different dimensions of parenting underpin favorable outcomes across the cultures (5). Several lines of evidence support this interpretation. For example, high parental control, consistently associated with poor outcomes in Western settings, is commonly not associated with negative outcomes in other cultural settings (24, 50–52). One explanation could be that some aspects of control, such as providing guidance through instructions, is perceived, in non-Western cultures, as a central parental responsibility and, consequently, children might experience these child-rearing practices as expressions of involvement and care as opposed to restriction of their autonomy and rejection (24, 50, 53–55). Another possibility is that, while in Western studies parental control is commonly associated with lack of warmth and other positive behaviors, in Asian settings there might be coexistence of positive affect expressed by parents and controlling behaviors, with the former buffering the relationship between high control and negative child outcomes (5, 35). Literature suggests that in Asian cultures, praise is used to encourage good behaviors and obedience and to promote interdependence with the social group as much as other forms of parenting aimed to direct child behaviors, such as verbal control (56–58). The context of this research is different because dyads are in a play situation rather than a disciplinary one, however our findings showing how Indian mothers are both more directive and more positive than UK mothers seem to support the premise that both strategies are dominant in Asian cultures. Looking at the transcripts, it appears clear that some positive comments made by mothers are more evaluative/expressing approval for behaviors (e.g., “Good boy/girl”, “Well done!”), while some others are more expression of admiration and affection (“You are so pretty”, “You are my love”). Future research might explore these differences further and the possible associations with other parenting behaviors and later child outcomes. If correct, the conclusion that the large differences that we observed reflect major differences in effective and supportive parenting across cultures would have implications for the identification of differential pathways to socialization and optimal development across cultures. Future studies should explore the association between different parenting styles and a variety of child development and educational outcomes.

### 4.3. Strengths and limitations

The strengths of our study included that families were selected at random from larger samples recruited from the general population during pregnancy, the observation procedures were the same in the UK and in India, the use of the percentage score for the parenting dimensions allowed controlling for level of verbosity, and transcripts were rated after cues to cultural context were removed. These methodological strengths may also have introduced limitations. First, the request to mothers to play as they would normally do with their children may not have meant the same thing to UK and Indian mothers, even though the piloting of the play task and previous studies confirmed that the procedure was acceptable and feasible in a urban Indian sample of mothers and infants (42). Second, 60% of the videos in the Indian sample were recorded at home instead of the lab and we cannot assume the location did not have any influence on maternal and child behavior. Third, rating from transcripts also introduced the limitation that coding depended on content of utterances rather than tone, and did not account for mothers’ non-verbal behaviors. In the case of mind-mindedness it was not possible to judge the appropriateness of the mothers’ comments for their infants’ behaviors; therefore, the group differences found in the use of mind-mindedness should be considered with caution, even though they seem to confirm the other few existing findings from cross-cultural studies conducted in Asia which found a lower prevalence in Asian samples compared to Wester ones (16–19). Fourth, both samples had high representations of low socio-economic families, so the findings may not generalize to more affluent contexts in either country. Finally, the two groups are not homogeneous in terms of socio-demographic characteristics (e.g., maternal age and education), which was due to differences between the UK and Indian populations (e.g., older, and better educated women in the UK sample compared to the Indian).

### 4.4. Future directions and clinical implications

The implications for future investigation are numerous. At the practical, clinical and policy level, we need to know more about variations in parenting practices across ethnic groups within Western settings, and how these may differ across generations, and similarly how these change with Westernization in non-Western settings. Research conducted in non-Western settings is growing, together with interest in studying cultural variations that exist within same countries due to the high presence of immigrant families and their children. Developing culturally appropriate parenting interventions based on knowledge of natural cultural variations in approach is fundamental to avoid transposing programs from one context to another which would result in delivering messages inconsistent with parents’ beliefs and their cultural norms. It should also increase the acceptability and effectiveness of such interventions. In terms of developmental processes, longitudinal studies are needed to test the key question of how different parenting dimensions are associated with later social, emotional, behavioral and educational outcomes across cultures (31). In line with emerging evidence linking specific aspects of parenting to specific types of psychopathologies, these studies need to examine whether these also vary by culture. Finally, future studies might benefit from the assessment of parenting styles of other caregivers especially in Asian settings where typically other family members care for children on a daily basis along with mothers.

## Data Availability

The datasets presented in this article are not readily available because of ethical constraints. Anonymised summary data may be made available to bona fide researchers on approval of an application for access. However, we do welcome formal approved collaborations for the analysis of data. Requests to access the datasets should be directed to hmsharp@liverpool.ac.uk.
