# Supplementary material for: Cross-cultural differences in early caregiving: levels of mind-mindedness and instruction in UK and India
Source: Front Child Adolesc Psychiatry. 2023 Aug 14;2:1124883. doi: 10.3389/frcha.2023.1124883 (PMC11748801; doi:10.3389/frcha.2023.1124883)
Supplement: Supplementary file 1 [file Table1.docx]

**Appendix Table 1 Spearman correlations between parental practices in the UK and Indian groups.**

|  | **Mind-minded**  **comments** | **Instructions** | **Control** |
| --- | --- | --- | --- |
| **UK sample** |  |  |  |
| Instructions | 0.142 |  |  |
| Control | -0.183 | .290^*^ |  |
| Positive comments | 0.160 | -0.094 | -0.028 |
| **Indian sample** |  |  |  |
| Instructions | 0.197 |  |  |
| Control | 0.029 | -0.012 |  |
| Positive comments | 0.133 | 0.010 | -0.163 |

**Appendix Table 2 – Total number of comments, and number of comments coded (total and for each parental dimension).**

|  | **Total number of utterances** | **Mind-mindedness**  **Total score** | **Instructions**  **Total score** | **Control**  **Total score** | **Positive comments**  **Total score** | **Total Comments coded** |
| --- | --- | --- | --- | --- | --- | --- |
| **UK sample** |  |  |  |  |  |  |
| Mean | 191.76 | 11.40 | 20.74 | 9.04 | 4.90 | 11.52 |
| Std. Deviation | 72.96 | 7.45 | 12.32 | 10.32 | 5.56 | 8.91 |
| **Indian sample** | |  |  |  |  |  |
| Mean | 266.36 | 5.02 | 71.72 | 62.30 | 31.00 | 42.51 |
| Std. Deviation | 154.61 | 6.46 | 56.88 | 52.88 | 20.81 | 34.26 |
